# Rare Variants in Inborn Errors of Immunity Genes Associated With Covid-19 Severity

**DOI:** 10.3389/fcimb.2022.888582

**Published:** 2022-05-27

**Authors:** Panhong Liu, Mingyan Fang, Yuxue Luo, Fang Zheng, Yan Jin, Fanjun Cheng, Huanhuan Zhu, Xin Jin

**Affiliations:** ^1^College of Life Sciences, University of Chinese Academy of Sciences, Beijing, China; ^2^Beijing Genomeics Institute At Shenzhen, BGI-Shenzhen, Shenzhen, China; ^3^Beijing Genomeics Institute In Singapore, BGI-Singapore, Singapore, Singapore; ^4^Department of Pediatrics, Union Hospital, Tongji Medical College, Huazhong University of Science and Technology, Wuhan, China; ^5^Department of Hematology, Union Hospital, Tongji Medical College, Huazhong University of Science and Technology, Wuhan, China; ^6^School of Medicine, South China University of Technology, Guangzhou, China

**Keywords:** Covid-19, inborn errors of immunity, gene-level tests, pathway-based analysis, PPI network analysis

## Abstract

Host genetic factors have been shown to play an important role in SARS-CoV-2 infection and the course of Covid-19 disease. The genetic contributions of common variants influencing Covid-19 susceptibility and severity have been extensively studied in diverse populations. However, the studies of rare genetic defects arising from inborn errors of immunity (IEI) are relatively few, especially in the Chinese population. To fill this gap, we used a deeply sequenced dataset of nearly 500 patients, all of Chinese descent, to investigate putative functional rare variants. Specifically, we annotated rare variants in our call set and selected likely deleterious missense (LDM) and high-confidence predicted loss-of-function (HC-pLoF) variants. Further, we analyzed LDM and HC-pLoF variants between *non-severe* and *severe* Covid-19 patients by (a) performing gene- and pathway-level association analyses, (b) testing the number of mutations in previously reported genes mapped from LDM and HC-pLoF variants, and (c) uncovering candidate genes *via* protein-protein interaction (PPI) network analysis of Covid-19-related genes and genes defined from LDM and HC-pLoF variants. From our analyses, we found that (a) pathways Tuberculosis (hsa:05152), Primary Immunodeficiency (hsa:05340), and Influenza A (hsa:05164) showed significant enrichment in *severe* patients compared to the *non-severe* ones, (b) HC-pLoF mutations were enriched in Covid-19-related genes in *severe* patients, and (c) several candidate genes, such as *IL12RB1*, *TBK1*, *TLR3*, and *IFNGR2*, are uncovered by PPI network analysis and worth further investigation. These regions generally play an essential role in regulating antiviral innate immunity responses to foreign pathogens and in responding to many inflammatory diseases. We believe that our identified candidate genes/pathways can be potentially used as Covid-19 diagnostic markers and help distinguish patients at higher risk.

## Introduction

Since December 2019, the coronavirus diseases 2019 (Covid-19) (Gorbalenya et al., 2020) caused by the SARS-CoV-2 virus (Severe Acute Respiratory Syndrome Coronavirus 2) (Zhu et al., 2020) has spread rapidly across the world. By January 2022, the ongoing SARS-CoV-2 pandemic has caused more than 360 million confirmed cases and more than 5 million deaths. Host genetic factors have been shown to play critical roles in the disease susceptibility and severity (Ellinghaus et al., 2020; COVID-19 Host Genetics Initiative, 2021; Pairo-Castineira et al., 2021; Shelton et al., 2021; Kousathanas et al., 2022). The Covid-19 Host Genetics Initiative (Covid-19 HGI, https://www.covid19hg.org/) is an international initiative to share the results of host genome-wide associations study (GWAS) meta-analysis of Covid-19 disease. The most recent Covid-19 HGI release 6 has reported 24 lead SNPs (P < 5e-8) mapped to nearly 136 genes, such as *LZTFL1*, *ABO*, *OAS1*, *DPP9*, and *IFNAR2* (COVID-19 Host Genetics Initiative, 2021). The estimated heritability of Covid-19 symptoms explained by these common variants was 6.5% (Pairo-Castineira et al., 2021). A twin study with participants from the TwinsUK cohort reported that 31% of phenotypic variance of predicted Covid-19 is due to host genetic factors (Williams et al., 2020). This leads to a large proportion of unexplained heritability (nearly 25%), commonly referred to as “missing heritability”. There is increasing evidence that rare variants also make a major contribution to missing heritability of many complex diseases and traits (Hunt et al., 2013; Zuk et al., 2014; Misawa et al., 2020).

Recently, the rare variants attracted researchers’ attention in explaining the missing heritability of Covid-19 disease. For example, Zhang et al. found that the rare predicted loss-of-function (pLoF) variants in the IRF7- and TLR3-dependent type I interferon (IFN) pathway were enriched in patients who developed risky Covid-19 (Zhang Q. et al., 2020). Smieszek et al. reported that pLoF variant in gene *IFNAR2* (c.966C>A/p.Y322X) might play a role not only in clinical manifestation of Covid-19 but also in the response to vaccination (Smieszek et al., 2021). In addition, multiple studies found that the pLoF variants in *TLR7* gene enriched in severely affected male patients, and the deficiency of TLR7 would impair innate immunity and increase severity of COVID-19 (van der Made et al., 2020; Asano et al., 2021; Fallerini et al., 2021; Mantovani et al., 2021). As previously reported, the rare variants were more likely to be functional and tended to have stronger effects on complex diseases (Gorlov et al., 2011). The study of genetic effects of rare variants is necessary to elucidate the severity of Covid-19.

To explore the genetic contributions of rare variants in Covid-19 patients with inborn errors of immunity (IEI) genes, we recruited and investigated nearly 500 hospitalized patients from Union Hospital of Tongji Medical College of Huazhong University of Science and Technology (abbr. Union Hospital) (Zhu et al., 2021). Based on patients’ genomic data and clinical information, we carried out three major analyses to investigate the effects of putative functional rare variants (LDM and HC-pLoF): (a) gene- and pathway-level tests of these rare variants between *severe* and *non-severe* patients; (b) examination of the significance of previously reported rare variants and genes in our dataset; and c) rare mutation accumulation analysis and PPI network analysis in only *severe* patients. From these analyses, we (a) identified candidate functional pathways that are responsible for innate immune disorders and respiratory diseases, such as Tuberculosis (hsa:05152), Primary Immunodeficiency (hsa:05340), and Influenza A (hsa:05164); (b) successfully replicated two Covid-19 associated SNPs (rs780744847 and rs541048548) mapped on genes *TLR3* and *ICAM3*, respectively; and (c) suggested several candidate genes, including *IL12RB1, TBK1, TLR3*, and *IFNGR2*, which might be involved in SARS-CoV-2 cell entry, host immune responses, and Covid-19 disease severity.

Until now, literature based on the Chinese population has replicated and discovered some Covid-19-associated common variants (Wang et al., 2020; Wu et al., 2021a; Wu et al., 2021b; Zhu et al., 2021), but genetic background of rare variants is currently insufficiently understood in the Chinese population. Our work is an effort to fill this gap. We hope that it will serve as a useful scientific reference to assess the genetic mechanism of rare variants in Covid-19 and advance our understanding of disease etiology.

## Materials and Methods

### Patient Recruitment and Quality Control

All subjects in this study were collected from the Union Hospital. We used PLINK 2.0 (Chang et al., 2015) to infer sex of individuals from SNP genotypes and VerifyBamID (Zhang F. et al., 2020) to assess DNA contamination level. Individuals were excluded if their inferred sex was inconsistent with that of clinically recorded. We also removed individuals with estimated contamination rates greater than 0.05. After sample quality control, there were 451 unrelated individuals with 159 mild and moderate patients, and 292 severe and critical patients. The severity classification criteria were made by the National Health Commission of P.R. China (Wu and McGoogan, 2020). We reclassified the mild/moderate patients as *non-severe* patients and the severe/critical patients as *severe* patients.

### Whole Genome Sequencing

Cell-free DNA (cf-DNA) was extracted from 200 ul plasma using MagPure Circulating DNA Kit following the manufacturer’s instructions. The 40 ul cf-DNA was extracted from each sample and used to create a library using MGIEasy Cell-free DNA Library Prep Kit according to the library preparation pipeline. Sequencing was conducted by the DNBSEQ platform (MGI, Shenzhen, China) to generate 100 bp paired-end reads. The mean sequencing depth was 17.8× for all samples.

### Genotype Calling

The blood samples of some patients were collected at different time points during hospitalization. To increase the average depth of study, sequence fastq files of each patient were merged together to generate one GVCF file by BWA (Li and Durbin, 2009) and Sentieon Genomics software (Freed et al., 2017). Joint variant calling was then performed on GVCF files of all participants using the Sentieon GVCFtyper algorithm. The resulting VCF file was used for subsequent genomic analyses.

After the application of excessHet (<54.69) filter, Variant Quality Score Recalibration (VQSR) was completed by using the Genome Analysis Toolkit (GATK version 4.1.2) (DePristo et al., 2011). Known variant files were downloaded from the GATK bundle. For SNP sets, we used SNPs of HapMap, 1000G_omin and 1000G_phase1 database as training sets, the SNPs of HapMap as true sets, and the SNPs of dbSNP as known sets. For indel sets, we used indel of Mills_and_1000G_gold_standard database as training and true sets. We used the annotations “DP”, “QD”, “MQRankSum”, “ReadPosRankSum”, “FS”, and “SOR” to train VQSR. Finally, we used a sensitivity threshold of 99.7% and 99% for the SNPs and INDELs, respectively, to define genotyped sites that passed VQSR filtration.

To improve the genotyping accuracy, we used the Beagle 4.0 software (Browning and Browning, 2016) to perform LD-based genotype refinement and imputation by taking genotype likelihoods as inputs. Low-quality variants with dosage imputation score DR2 < 0.3 were filtered out.

### Principal Component Analysis

Principal component analysis (PCA) (Pearson, 1901) was performed using a subset of autosomal bi-allelic SNPs by applying PLINK 2.0 (Chang et al., 2015). Several restrictions were applied to select SNPs for PCA analysis, including keeping SNPs with minor allele frequency (MAF) ≥ 5%, Hardy–Weinberg Equilibrium P ≥ 1e-6, and removing one of a pair of SNPs if the LD was greater than 0.5 (in a window of 50 SNPs with a step of 5 SNPs). To select an adequate number of significant PCs in the association analysis, we performed hypothesis testing for each eigenvalue by using software Eigenstrat (Price et al., 2006).

### Functional Annotation

We annotated rare variants (MAF < 0.5%) in our final call set by using the Ensembl Variant Effect Predictor (VEP, build 103, GRCh38) (McLaren et al., 2016) with default parameters. The databases for annotation included dbSNP (Sherry et al., 2001), gnomAD (Karczewski et al., 2020), and 1000 Genomes Project (Clarke et al., 2012). In addition, we used Combined Annotation Dependent Depletion (CADD) score to predict missense variants that had potential effects on protein function. The CADD score was annotated by CADD plug-in (Kircher et al., 2014). Missense variants with CADD score > MSC (Mutation Significance Cut-off) score (95% confidence interval) (Itan et al., 2016) were predicted as likely deleterious missense (LDM) variants. We also used LOFTEE (Karczewski et al., 2020) plug-in to identify high-confidence pLoF (HC-pLoF) for stop-gained, frameshift, and splice site disrupting variants. Finally, we focused on the LDM and HC-pLoF variants in the subsequent analyses.

### Rare Variants Analyses

To investigate the cumulative effects of multiple rare variants, we performed gene-based association analysis using KGGSeq 1.0 (Li et al., 2017) with the sequence kernel association test (SKAT) (Wu et al., 2010), the Optimized SKAT (SKAT-O) (Lee et al., 2012), and Burden test. We used the binary collapsing method (burden test) implemented in KGGseq. We further carried out pathway-based analysis by testing the Kyoto Encyclopedia of Genes and Genomes (KEGG) gene sets (Kanehisa and Goto, 2000). The adjusted covariates included age, sex, and the top six principal components. We defined the suggestive significance threshold for gene-based association test as 1e–6 and for pathway-based association test as 0.05.

We also focused on 24 type I IFN genes (denoted as IFN-genes) that were found as an enrichment in a life-threatening Covid-19 study (Zhang Q. et al., 2020; Kosmicki et al., 2021) and 136 genes located in 50 kb of lead SNPs reported by the Covid-19 Host Genetics Initiative (release 6, denoted as HGI-genes) (COVID-19 Host Genetics Initiative, 2021) (**Supplementary Table S1**).The mutation accuracy of variants in these 159 known candidate genes (one overlap between 24 IFN-genes and 136 HGI-genes) was manually checked by using Samtools 1.10 (Danecek et al., 2021).

Finally, we performed an analysis of rare variant accumulation in genes identified by two approaches. The first approach detected genes if one variant met the following two conditions: (a) the mutations occurred in only *severe* patients, and (b) the variant harbored no less than three effect allele counts. We denoted these genes as “individual variant-driven” genes. The second approach determined genes if (a) all mutations in the gene occurred in only *severe* patients, and (b) the total number of mutations in the gene is at least three. We denoted these genes as “all variant-driven” genes. We note that genes identified by the two methods may have some overlap. Each of the two gene sets was then used for PPI network analysis with the above 159 known candidate genes. We used the STRING version 10.5 (Search Tool for the Retrieval of Interacting Genes/Proteins) (Szklarczyk et al., 2019) to build the PPI network. The minimum required interaction score to the highest confidence was set to 0.900.

## Results

### Participant Characteristics

In this study, participants included 451 Covid-19 patients aged 23 to 97 years old and all declared Han Chinese population. In **Table 1**, we provide participant characteristics for *non-severe* and *severe* patients, respectively. A total of 159 (35.25%) and 292 (64.75%) patients were grouped as *non-severe* and *severe*, respectively. The same as previously reported (Cummings et al., 2020; Yang et al., 2020), patients with older age (*severe*: an average of 64 years old vs. *non-severe*: an average of 58 years old, t-test *p* = 4.6e-05) and men (*severe* 52.74% vs. *non-severe* 42.14%, Fisher’s exact test *p* = 0.04) were at a higher risk of developing severe symptoms.

**Table 1:** Participant characteristics.

	Number, n (%)	Men, n (%)	Age, average (sd)	Depth, average (sd)
**All patients**	451			21.67
**Non-severe**	159 (35.25%)	67 (42.14%)	58.33 (14.62)	19.04 (8.94)
**Severe**	292 (64.75%)	154 (52.74%)	64.11 (13.31)	23.1 (10.43)

### Data Quality

**Figure 1:**
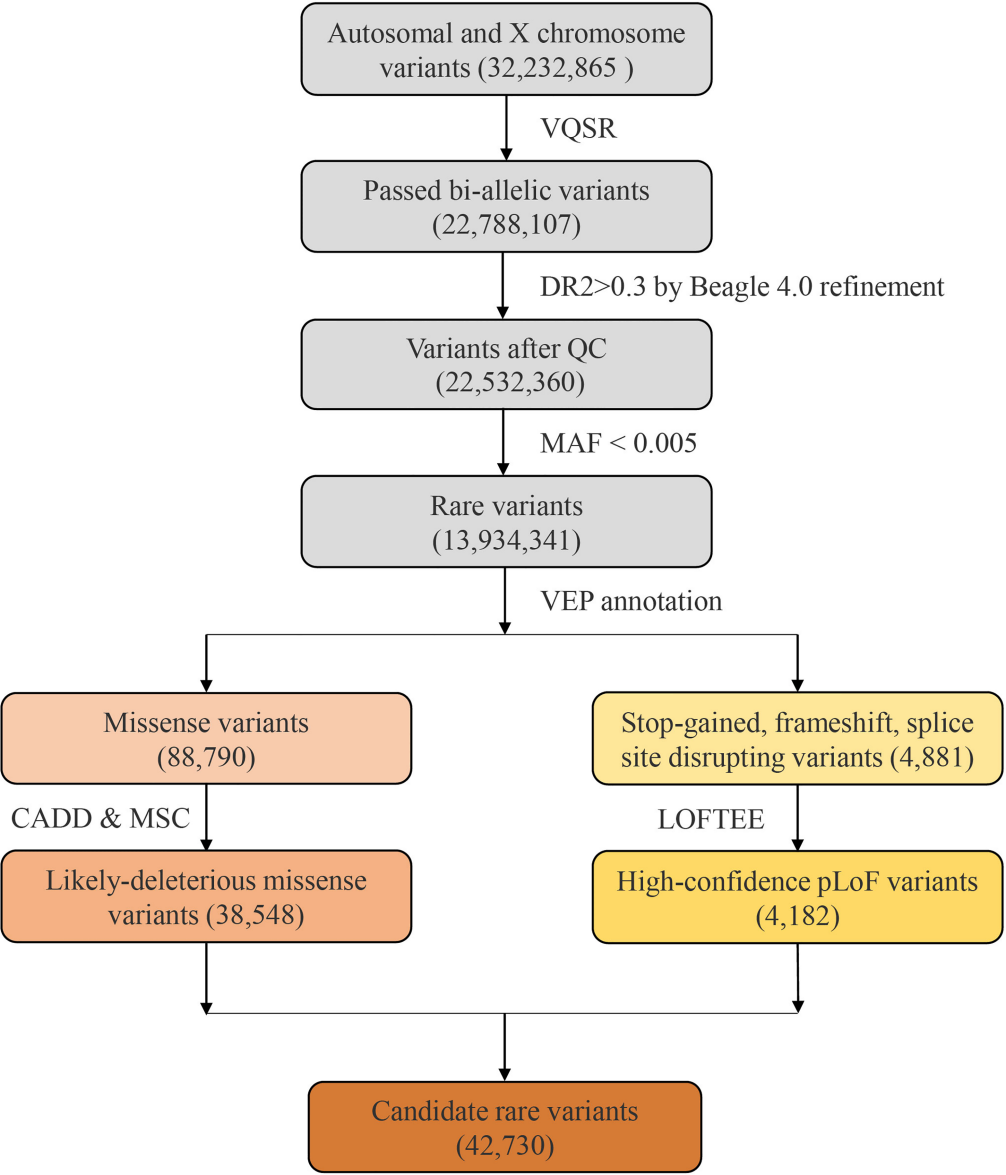
The flow diagram of rare variants analysis. A total of 32,232,865 variants were identified from the 451 Covid-19 patients with whole genome sequencing. After filtering by VQSR and MAF, 13,934,341 rare variants were annotated by VEP, and 42,730 candidate variants were included.

**Figure 2:**
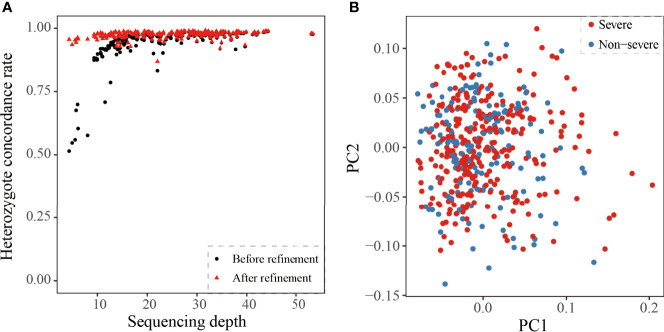
Quality estimate of the cohort. **(A)** Heterozygote concordance rate vs. sequencing depth for 218 array-genotyped individuals. The black point and red triangle represent one sample before and after refinement. **(B)** PCA of 159 non-severe and 292 severe Covid-19 patients. The red and blue represent the severe or non-severe patients.

After quality control, the dataset consisted of 22,107,585 and 680,522 variants from autosomes and X chromosome, respectively (**Figure 1**). Then we compared chip array sequencing results with genotype after LD-based refinement by Beagle 4.0 on 218 individuals. The heterozygote concordance rate increased from an average of 94.4% to 97.4%, and the improvement is more dramatic for samples with lower sequencing depth (**Figure 2A**). After filtering in variants by imputation score DR2 > 0.3, the final dataset for further analyses had a total of 22,532,360 variants, and the PCA on 575,888 autosome SNPs detected no outlier samples (**Figure 2B**).

### Rare Variants Statistics

After filtering by MAF, we obtained a total of 13,934,341 rare variants for VEP annotation. Among the resulting annotations, there were 88,790 missense variants and 4881 pLoF variation (including stop-gained, frameshift, and splice site disrupting variants). Damaging effects of these missense and pLoF variants were then predicted by CADD and LOFTEE plug-in, respectively. About 43.41% missense variants were predicted as LDM variants (38,548) and 85.68% pLoF variants were predicted as HC-pLoF variants (4182). Thus, in total, 42,730 predicted likely damaging variants were applied for further analysis. For both LDM variants and HC-pLoF variants, we first tested the difference of their numbers between *non-severe* and *severe* patients and found no significant difference (**Supplementary Table S2**).

### Gene- and Pathway-Level Analysis of Rare Variants

The gene-level analysis of rare variants was performed between *severe* and *non-severe* patients *via* KGGSeq. We performed the gene-based tests for genes mapped by all 42,730 rare variants, 38,548 LDM variants, and 4182 HC-pLoF variants, respectively. The gene-based analyses did not identify genes that passed the significance threshold of 1e-6 (**Supplementary Figures S1A–C**).

Furthermore, we leveraged the biological knowledge that sets of genes acting together in pathways. In total, we tested 307 KEGG pathways and detected Tuberculosis (hsa:05152, P-burden = 0.036) between *severe* and *non-severe* patients on LDM and HC-pLoF variants (**Supplementary Table S3**). Tuberculosis (TB) is an airborne infectious disease caused by Mycobacterium tuberculosis (Mtb). It first attacks the lungs, then other parts of the body through the circulatory system. This transmission characteristic is remarkably similar to that of Covid-19. As previously reported, Tuberculosis pathway was significant with acute respiratory distress syndrome and lung injury in mice and humans (Sweeney et al., 2017). The TB/Covid-19 Global Study Group observed a phenomenon that TB and SARS-CoV-2 might be co-infected, that is, TB was often diagnosed concurrently or after Covid-19 infection and the co-infection might account for increased case fatality rate (The TB/COVID-19 Global Study Group, 2021). Our finding brought up a possible explanation that patients with rare mutations enriched in Tuberculosis pathway were more likely to develop severe Covid-19 symptoms. More details about this candidate pathway, including the contributed genes, the corresponding gene-based results, and the number of carriers in cases and controls, were provided in **Supplementary Table S4**. When focusing on only pLOF variants enriched on KEGG, two significant pathways highlighted: Primary immunodeficiency (hsa:05340, P-burden = 0.014) and Influenza A (hsa:05164, P-burden = 0.021) (**Supplementary Table S2**). Primary immunodeficiencies (PID) are a group of potentially serious disorders that can cause increased susceptibility to severe infections, autoimmune diseases, and malignancy. Several studies revealed that patients with PID displayed higher morbidity and mortality from Covid-19 (Hunt et al., 2013; Zuk et al., 2014; Trinder et al., 2020; Kousathanas et al., 2022). Influenza is an infectious respiratory disease caused by influenza virus. Bibert et al. observed that gene pathways involved in the detection of Influenza A overlapped with those involved in the detection of SARS-CoV-2 virus (Bibert et al., 2021). In these two biological pathways, three functional genes, *IKBKG*, *IRF7*, and *IFNAR1*, were previously identified to have an effect on Covid-19 severity (Zhang Q. et al., 2020). More details about these two potentially functional pathways are provided in **Supplementary Tables S5 and S6**.

### Tested on 159 Candidate Genes

In addition to uncovering unknown possibly associated genes or pathways, we also tested 159 previously reported candidate genes, with 24 in the IFN pathway (Zhang Q. et al., 2020; Kosmicki et al., 2021) and 136 located within 50 kb of significant common variants in the Covid-19 HGI (COVID-19 Host Genetics Initiative, 2021). Specifically, we focused on LDM and HC-pLOF variants to aggregate potential effects of rare variants. For missense variants in both IFN- and HGI-genes, we did not detect significant difference between *severe* and *non-severe* patients.

In the 24 IFN-genes, we found one HC-pLoF variant rs780744847 (c.1180C>T/p.R394*) on *TLR3* mutated in only *severe* patients but no mutations in *non-severe* patients. It was reported that the *TLR3* deficiency may lead to increased incidences of viral infections and impair the production of type I IFN throughout SARS-CoV-2 infection (Zhang Q. et al., 2020). Moreover, mutations of inborn errors of TLR3-dependent type I IFN immunity more often occurred in highly critical patients than in mild patients and healthy controls.

For the 136 HGI-genes, we found that the number of HC-pLoF mutations occurred in the *severe* group was more than that of the *non-severe* groups (16 in *severe* and 2 in *non-severe* patients, Fisher’s exact test p = 0.043) (**Table 2**). We also detected a HC-pLoF variant rs541048548 (c.1053del/p.A352fs) on *ICAM3* only mutated in *severe* patients. The gene *ICAM3* played an important role in the immunopathogenesis of SARS virus (Chan et al., 2007) and had been reported that its expression was downregulated in asymptomatic Covid-19 cases compared with symptomatic patients (Masood et al., 2021).

**Table 2:** The pLoF variants identified in Covid-19 patients in 159 candidate genes.

Gene	SNP	Variant annotation	HGVSc/HGVSp	Genotype	Sample ID	Sex	Age range	Phenotype	Category
*TLR3*	rs780744847	Stop gained	c.1180C>T/p.Arg394Ter	Het	U312	F	70-79	Severe	IFN-genes
*THBS3*	rs748584696	Stop gained	c.853C>T/p.Arg285Ter	Het	U088	F	80-89	Severe	HGI-genes
*THBS3*	chr1_155206198_A_C	Splice donor variant	c.286+2T>G	Het	U359	F	70-79	Severe	HGI-genes
*TAC4*	rs372635644	Splice acceptor variant	c.124-1G>A	Het	U429	F	60-69	Severe	HGI-genes
*TYK2*	rs770927552	Frameshift variant	c.209_212del/p.Cys70SerfsTer21	Het	U422	F	80-89	Severe	HGI-genes
*C6orf15*	chr6_31112292_C_T	Splice acceptor variant	c.68-1G>A	Het	U107	M	60-69	Severe	HGI-genes
*CAT*	rs777641795	Splice donor variant	c.1195+1G>A	Het	U012	M	40-49	Non-severe	HGI-genes
*CDH15*	chr16_89179469_C_G	Stop gained	c.96C>G/p.Tyr32Ter	Het	U174	F	50-59	Severe	HGI-genes
*CDSN*	chr6_31116133_G_GA	Frameshift variant	c.1481dup/p.Cys496LeufsTer20	Het	U021	F	50-59	Non-severe	HGI-genes
*ICAM3*	rs541048548	Frameshift variant	c.1053delC/p.Ala352ArgfsTer11	Het	U225	M	70-79	Severe	HGI-genes
*ICAM3*	rs541048548	Frameshift variant	c.1053delC/p.Ala352ArgfsTer11	Het	U047	F	70-79	Severe	HGI-genes
*MYDGF*	rs745851558	Splice donor variant	c.174+1G>T	Het	U071	M	70-79	Severe	HGI-genes
*PLEKHA4*	chr19_48853718_CA_C	Frameshift variant	c.1289delT/p.Leu430ArgfsTer4	Het	U261	F	60-69	Severe	HGI-genes
*PLEKHA4*	chr19_48853720_GCCGGT_G	Frameshift variant	c.1283_1287delACCGG/p.Asp428AlafsTer76	Het	U261	F	60-69	Severe	HGI-genes
*PPP1R15A*	rs768756506	Frameshift variant	c.1535_1536delAT/p.Tyr512CysfsTer14	Het	U309	M	60-69	Severe	HGI-genes
*PSORS1C2*	rs79153019	Frameshift variant	c.281delC/p.Pro94LeufsTer35	Het	U075	M	60-69	Severe	HGI-genes
*PSORS1C2*	rs79153019	Frameshift variant	c.281delC/p.Pro94LeufsTer35	Het	U150	M	60-69	Severe	HGI-genes
*PSORS1C2*	rs79153019	Frameshift variant	c.281delC/p.Pro94LeufsTer35	Het	U144	M	60-69	Severe	HGI-genes
*TULP2*	chr19_48881045_T_TC	Frameshift variant	c.1528_1529insG/p.Gln510ArgfsTer17	Het	U176	F	70-79	Severe	HGI-genes

### Mutation Accumulation Analyses

In the mutation accumulation analysis, we first investigated whether there were potentially functional mutations unique to *severe* patients. We filtered in rare variants mutated in only *severe* patients and with minor allele count (MAC) greater than or equal to three. This resulted in 756 rare variants mapped to 700 genes. Among these variants, we observed a very rare mutation rs777044791 in gene *CCR3* at locus 3p21.31 (**Table 3**). The physical distance between rs777044791 and rs11385942 is 0.43 MB (GRCh38), a distance typically flanked into the same genomic region (Casto and Feldman, 2011). The variant rs11385942 is a common variant located at locus 3p21.31 in European populations and was first identified to be associated with respiratory failure due to Covid-19 from GWAS analysis in Italian and Spanish populations (Ellinghaus et al., 2020). This finding was repeated in other studies based on European populations (COVID-19 Host Genetics Initiative, 2021; Pairo-Castineira et al., 2021; Shelton et al., 2021), verifying its effects on Covid-19 disease. In the Chinese population, common variant studies at this locus did not replicate significance (Wang et al., 2020; Wu et al., 2021b; Zhu et al., 2021), and no rare variant studies had been conducted. Our work raised a possibility that SNPs in locus 3p21.31 might also play an important role in Covid-19 severity in the Chinese population.

**Table 3:** The comparison of allele frequency for two loci.

	rs777044791	rs11385942
CHROM	chr3	chr3
POS (hg38)	46,266,186	45,834,967
ALT	T	GA
REF	C	G
Variant annotation	Missense variant	Intron variant
***Allele frequency***		
Severe (N = 292)	0.005	0
Non-severe (N = 159)	0	0
ChinaMAP	0.002	0.004
1000G_EAS	0	0.005
1000G_EUR	0	0.0805
1000G_SAS	0	0.296
1000G_AFR	0	0.053
gnomAD_EAS	0.0005	0.0006

**Figure 3:**
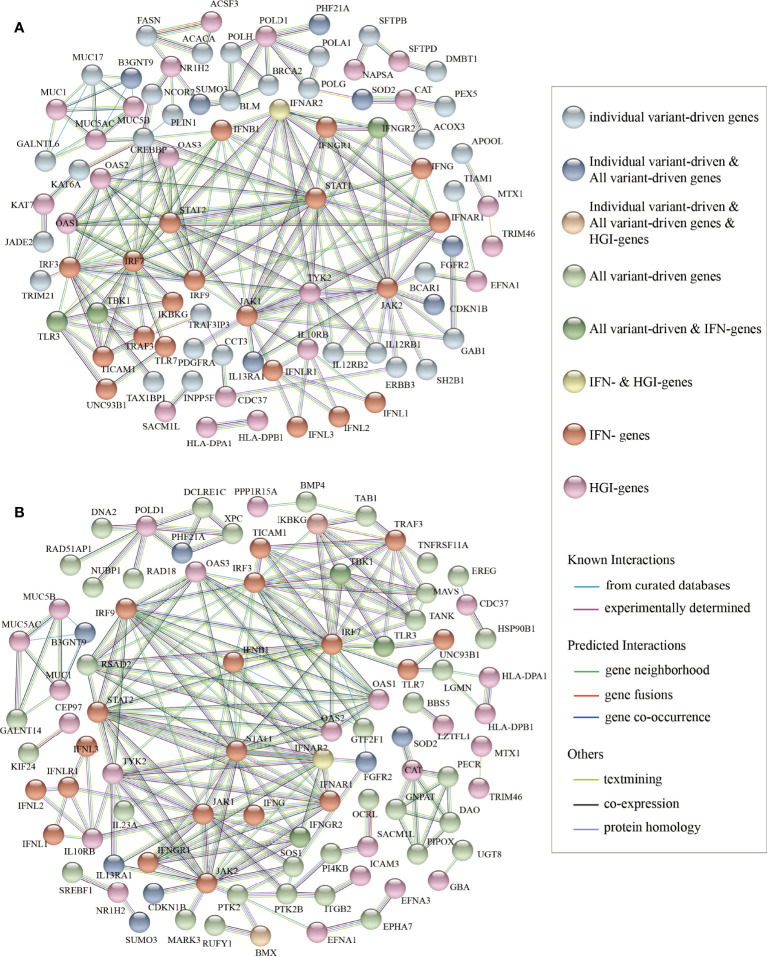
The results of protein-protein interaction network analysis. The plot of the PPI network **(A)** between the “individual variant-driven” genes with the candidate genes, and **(B)** between the “all variant-driven” genes with the candidate genes.

Then, we performed PPI network analysis for the 700 “individual variant-driven” genes with the 159 known genes (**Figure 3A**). From the results, we found two candidate genes *IL12RB1* and *TRAF3IP3* that had extensive interactions with IFN- and HGI-genes. Gene *IL12RB1* (Interleukin 12 Receptor Subunit Beta 1) encodes a type I transmembrane protein that binds to interleukin-12 (IL12) and is involved in IL12 transduction. Mutations in *IL12RB1* damage the development of IL17-producing T lymphocytes and increase the susceptibility to Salmonella and mycobacterial infections (van de Vosse et al., 2013). Our PPI network analysis indicated that *IL12RB1* and *TYK2* had experimentally determined interactions, which were compiled from a set of public databases and were more likely to be credible (Zhang et al., 2013). Gene *TYK2* had been previously identified to be associated with Covid-19 critical illness (Pairo-Castineira et al., 2021), implying the potential effects of *IL12RB1* to the aggravation of Covid-19. The gene *TRAF3IP3* (TRAF3 Interacting Protein 3) encodes a protein that plays essential roles in both innate and adaptive immunity. Knockout mouse experiments of this gene observed a decrease in white blood cell count in males and an increased susceptibility to bacterial infection (Gardin and White, 2011). In our results, TRAF3IP3 was experimentally determined with protein TRAF3 encoded by gene *TRAF3*, which was included in a newly created pathway “Activation of NLRP3 inflammasome by SARS-CoV-2” (WP4876) (Siu et al., 2019). In response to viral infection, *TRAF3IP3* bridges *TRAF3* and *MAVS* leading to interferon production, indicating it’s probably strong relationship with Covid-19 disease.

We also performed PPI network analysis for the 778 “all variant-driven” genes with the 159 known genes (**Figure 3B**), from which three genes, *TBK1*, *TLR3*, and *IFNGR2* were highlighted. Specifically, *TBK1* (TANK Binding Kinase 1) encodes a protein that plays important roles in antiviral innate immune response and in regulating inflammatory response to foreign agents (Fitzgerald et al., 2003; Mori et al., 2004). A previous study observed colocalization of *TBK1* with the M protein of SARS-CoV-2, which might hinder the dsRNA-induced IFN production at the step or upstream of *TBK1* (Zheng et al., 2020). The gene *TLR3* (Toll Like Receptor 3) encodes a member of the TLR family that plays a primary role in recognition of pathogen and innate immunity activation. It recognizes dsRNA participated in multiple viral infections and induces type I IFNs production (Kawai and Akira, 2007). The gene *IFNGR2* (Interferon Gamma Receptor 2) encodes the non-ligand-binding beta chain of the gamma interferon receptor. A recent study revealed a new set of genes that upregulated in severe Covid-19 patients compared to mild ones, probably triggered by *IFNGR1* and *IFNGR2* (Henriques-Pons et al., 2021).

In summary, our mutation accumulation analyses and PPI network analyses suggested that *IL12RB1*, *TRAF3IP3*, *TBK1*, and *TLR3* and *IFNGR2* are key regions in *severe* Covid-19 patients compared with *non-severe*, implying their functions and associations with Covid-19 severity.

## Conclusion

In our study, we have uncovered several functional pathways associated with Covid-19 severity, for example, Tuberculosis (hsa:05152), Primary Immunodeficiency (hsa:05340), and Influenza A (hsa:05164). These pathways are all responsible for innate immune disorders and respiratory diseases, highlighting the importance of host innate immune system against Covid-19. Our mutation accumulation analysis and PPI network analysis suggested several novel candidate genes in the Chinese population, including *IL12RB1*, *TBK1*, *TLR3*, and *IFNGR2*. These genes are potentially involved in SARS-CoV-2 cell entry, host immune responses, and finally influencing Covid-19 severity.

On one hand, our work filled the gap of IEI analysis in Covid-19 patients in the Chinese population; on the other hand, we replicated several Covid-19-associated genes first identified from a European population and also discovered some candidate genes specific to the Chinese population.

## Discussion

SARS-CoV-2 is a strain of coronavirus and is highly pathogenic and transmissible. After exposure to the virus, ordinary people may not develop noticeable symptoms or develop mild to moderate symptoms, while people with IEI tend to suffer severe and critical symptoms, or even death (Zhang S.-Y. et al., 2020). There is increasing evidence that the host genetic variants in genes related to immunodeficiency or inflammasomes might attribute to Covid-19 clinical manifestations (Elhabyan et al., 2020). Many clinical drug treatments for Covid-19 were cultivated from this finding, including type I IFNs (e.g., IFN-α1b), TNF inhibitors, anti-IFN-γ antibodies, JAK1 inhibitors, and STAT1 inhibitors (Ku et al., 2021).

In this work, we carried out the first study of rare variants in IEI genes associated with Covid-19 severity in the Chinese population. The identified functional candidate pathways Tuberculosis, Primary Immunodeficiency, and Influenza A were previously known to be part of antiviral immune responses and viral eradication, and we discovered their potential influences in Covid-19. We also suggested several putative genetic regions probably involved in susceptibility and severity of Covid-19, including genes *IL12RB1*, *TRAF3IP3*, *TBK1*, *TLR3*, and *IFNGR2*. Our work highlighted the importance of rare IEI in Covid-19 patients and people with IEI defects are more likely to be infected with SARS-CoV-2 and to develop severe symptoms. Thus, we appeal more studies on IEI in both the Chinese population and other populations to pinpoint causal genes of Covid-19 severity and finally help identify those patients at higher risk.

Despite the many compelling and significant findings of our work, there are still a few limitations to be noted. First, the sample size we used is relatively small, and the limited sample size limits the statistical power for identifying rare variants. More studies with large sample sizes are demanded to validate our results and uncover more candidate variants. Second, our work has suggested several candidate genes and pathways potentially related to Covid-19 severity, yet unfortunately, due to resources limitations, we are unable to perform web-lab experiments and verify gene functions at this stage. More persuasive experimental designs are needed to investigate how these candidate genes/pathways affect disease progression.

Covid-19 is assessed as a complex infectious disease and affected many risk factors. Symptoms of Covid-19 are highly variable, ranging from unnoticeable to severe and even death. The host genetic background is only partly responsible for the phenotypic heterogeneity. In recent years, multi-omics studies have proven a powerful and successful strategy to provide a broader perspective in understanding disease development and biological phenomena. Several multi-omics analyses of Covid-19 have been proposed to integrate multiple “omes” data to unravel disease mechanisms at multiple omics levels (Su et al., 2020; Montaldo et al., 2021; Overmyer et al., 2021; Stephenson et al., 2021; Wu et al., 2021a). The integrative analyses of rare genome and other “omes” data (e.g., proteome, transcriptome, epigenome, metabolome, and microbiome) may inspire us to discover new risk factors for severe Covid-19 disease.

## Data Availability Statement

The data that support the findings of this study have been deposited into CNGB Sequence Archive (CNSA) (Guo et al., 2020) of China National GeneBank DataBase (CNGBdb) (Guo et al., 2020) with accession number CNP0002853.

## Ethics Statement

The studies involving human participants were reviewed and approved by Medical Ethics Committee of Union Hospital, Tongji Medical College, Huazhong University of Science and Technology. The patients/participants provided their written informed consent to participate in this study.

## Author Contributions

XJ and HZ conceived the study, designed the research program, and managed the project. PL and HZ performed the statistical analyses. MF advised on statistical methods. PL and HZ wrote the manuscript. FC, FZ, and YJ collected the samples. YL finished the laboratory processing and data acquisition. All authors participated in revising the manuscript. All authors read and approved the final manuscript.

## Funding

This study was supported by National Natural Science Foundation of China (No. 31800765, 32171441, 32000398), Natural Science Foundation of Guangdong Province, China (2017A030306026), and Guangdong-Hong Kong Joint Laboratory on Immunological and Genetic Kidney Diseases (2019B121205005).

## Conflict of Interest

Authors PL, MF, YL, HZ, and XJ were employed by BGI-Shenzhen.

The remaining authors declare that the research was conducted in the absence of any commercial or financial relationships that could be construed as a potential conflict of interest.

## Publisher’s Note

All claims expressed in this article are solely those of the authors and do not necessarily represent those of their affiliated organizations, or those of the publisher, the editors and the reviewers. Any product that may be evaluated in this article, or claim that may be made by its manufacturer, is not guaranteed or endorsed by the publisher.

## Supplementary Material

Supplementary Figure 1 Results of gene-based association tests of rare variants. **(A)**. The QQ plots of gene-based association analyses between severe and non-severe patients for **(A)** 42,730 candidate rare variants; **(B)** 38,548 rare likely-deleterious missense variants, and **(C)** 4182 high-confidence pLoF variants. The color represents different association methods integrated in KGGseq, including SKAT test (in blue), SKAT-O (in red), and burden test (in orange).
