# Supplementary figures and images for: Rare Variants in Inborn Errors of Immunity Genes Associated With Covid-19 Severity
Source: Front Cell Infect Microbiol. 2022 May 27;12:888582. doi: 10.3389/fcimb.2022.888582 (PMC9184678; doi:10.3389/fcimb.2022.888582)

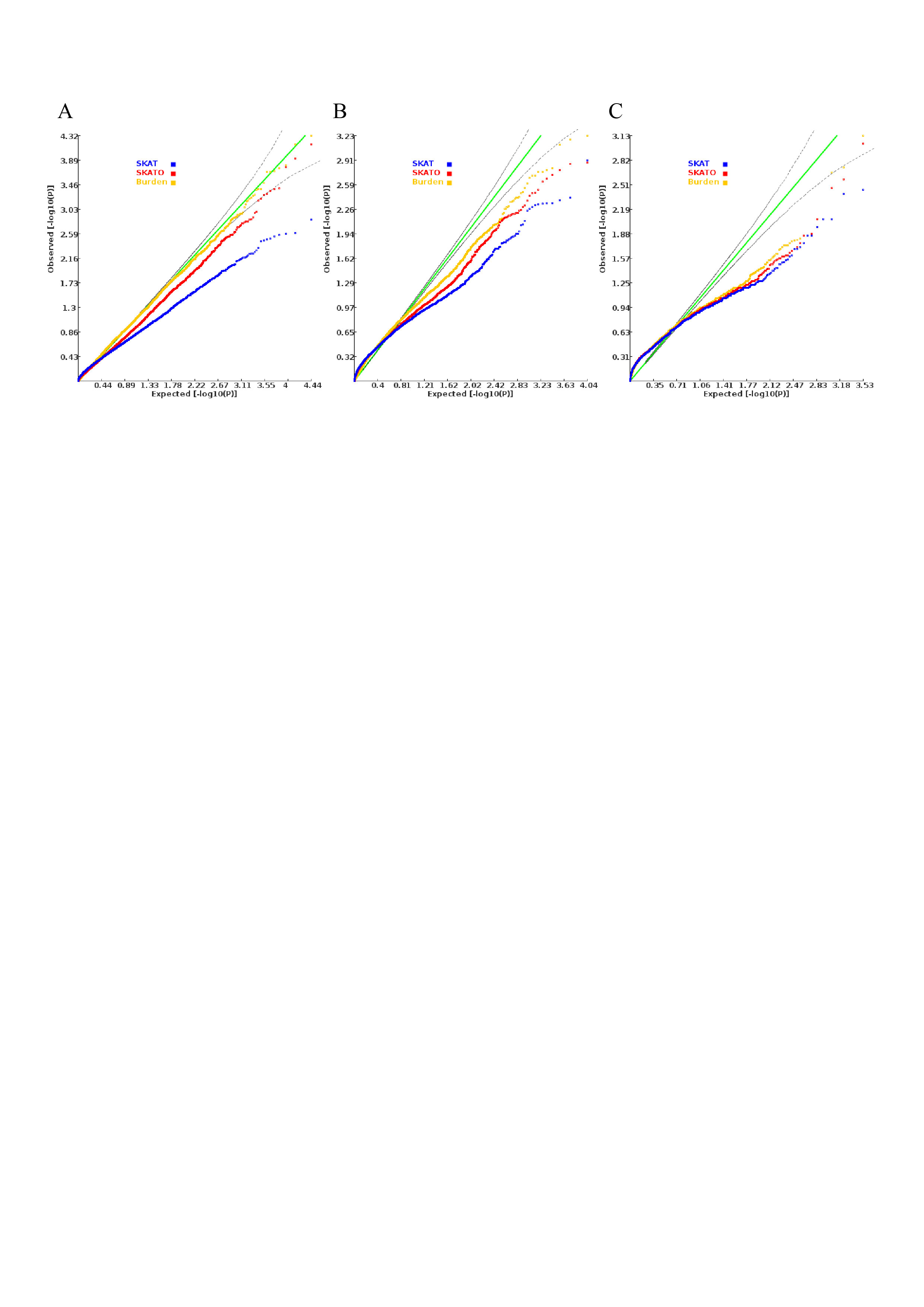

Supplement: Supplementary Figure 1 — Results of gene-based association tests of rare variants. (A). The QQ plots of gene-based association analyses between severe and non-severe patients for (A) 42,730 candidate rare variants; (B) 38,548 rare likely-deleterious missense variants, and (C) 4182 high-confidence pLoF variants. The color represents different association methods integrated in KGGseq, including SKAT test (in blue), SKAT-O (in red), and burden test (in orange). [file Image_1.jpeg]
